# The serine protease prostasin (PRSS8) is a potential biomarker for early detection of ovarian cancer

**DOI:** 10.1186/s13048-016-0228-9

**Published:** 2016-03-31

**Authors:** Ayala Tamir, Anju Gangadharan, Sakshi Balwani, Takemi Tanaka, Ushma Patel, Ahmed Hassan, Stephanie Benke, Agnieszka Agas, Joseph D’Agostino, Dayoung Shin, Sunghoon Yoon, Andre Goy, Andrew Pecora, K. Stephen Suh

**Affiliations:** The Genomics and Biomarkers Program, The John Theurer Cancer Center, Hackensack University Medical Center, D. Jurist Research Building, 40 Prospect Avenue, Hackensack, NJ 07601 USA; Stephenson Cancer Center, University of Oklahoma Health Science Center, Oklahoma city, OK 73104 USA; Clinical Divisions, John Theurer Cancer Center, Hackensack University Medical Center, Hackensack, NJ 07601 USA

**Keywords:** Prostasin, PRSS8, Early detection, Ovarian cancer, Biomarkers, Diagnostic, Serum

## Abstract

**Background:**

Ovarian cancer (OVC) is the deadliest of all gynecologic cancers, primarily as a consequence of asymptomatic progression. The complex nature of OVC creates challenges for early detection, and there is a lack of specific and sensitive biomarkers suitable for screening and detecting early stage OVC.

**Methods:**

Potential OVC biomarkers were identified by bioinformatic analysis. Candidates were further screened for differential expression in a library of OVC cell lines. OVC-specific overexpression of a candidate gene, PRSS8, which encodes prostasin, was confirmed against 18 major human cancer types from 390 cancer samples by qRT-PCR. PRSS8 expression profiles stratified by OVC tumor stage-, grade- and subtype were generated using cDNA samples from 159 OVC samples. Cell-specific expression and localization of prostasin was determined by immunohistological tissue array analysis of more than 500 normal, benign, and cancerous ovarian tissues. The presence of prostasin in normal, benign, and OVC serum samples was also determined.

**Results:**

Gene expression analysis indicated that PRSS8 was expressed in OVC at levels more than 100 fold greater than found in normal or benign ovarian lesions. This overexpression signature was found in early stages of OVC and was maintained in higher stages and grades of OVC. The PRSS8 overexpression signature was specific for OVC and urinary bladder cancer among 18 human cancer types. The majority of ovarian cell lines overexpressed PRSS8. In situ hybridization and histopathology studies of OVC tissues indicated that overexpression of prostasin was largely localized to tumor epithelium and was absent in neighboring stroma. Significantly higher levels of prostasin were found in early stage OVC serum samples compared to benign ovarian and normal donor samples.

**Conclusions:**

The abundant amounts of secreted prostasin found in sera of early stage OVC can potentially be used as a minimally invasive screening biomarker for early stage OVC. Overexpression of PRSS8 mRNA and high levels of prostasin in multiple subtypes of early stage ovarian tumors may provide clinical biomarkers for early detection of OVC, which can potentially be used with CA125 and HE4.

**Electronic supplementary material:**

The online version of this article (doi:10.1186/s13048-016-0228-9) contains supplementary material, which is available to authorized users.

## Background

Ovarian cancer (OVC) is the fifth most common cause of cancer-related death in women [1], and results in over 14,000 deaths annually in the US. It is considered to be the most lethal malignancy of the female reproductive system, largely because it is usually diagnosed at an advanced stage [2]. While the overall 5-year survival for patients in different stages of this malignancy is 45 %, the survival rate is as high as 90 % when the disease is diagnosed at an early stage (stage I/II) compared to only 11 % when diagnosed at an advanced stage. Unfortunately, because of the asymptomatic nature of the disease, nearly 80 % of new cases of OVC are diagnosed at advanced stages (III/IV). Thus, early detection of the disease is critical to reducing mortality. In addition to asymptomatic progression, early stage diagnosis has been difficult to achieve because OVC exhibits a wide range of morphological, clinical, and genetic variations during the course of tumor progression [2, 3]. Robust biomarkers that are sensitive and specific for OVC are needed for effectively screening the general population. CA125 has been used for years as a gold standard for disease monitoring and for assessing relapse or response to treatment. However, CA125 has low specificity for OVC [4] as well as less than optimal ability to detect all types of OVC; therefore, CA125 is not an optimal biomarker for early detection. More recently, the diagnostic value of CA125 has been shown to be improved when used in combination with other markers, including CA19-9, MCSF, OVX1, and HE4 [5, 6]. Additionally, the OVA1 blood test is an FDA-cleared test that helps evaluate an ovarian mass for malignancy prior to surgery. Apart from evaluating levels of CA125 and Beta-2 microglobulin, which are expected to be up-regulated in malignant conditions; the test also measures levels of apolipoprotein A1, prealbumin, and transferrin, which are expected to be down-regulated. In 2011, the FDA approved the use of blood tests for HE4 and CA125 with the Risk of Ovarian Malignancy Algorithm (ROMA), which demonstrated higher accuracy in determining risk in pre- and post-menopausal women. Additional tests such as OVACheck, which is based on proteomic technology, and OvaSure, which includes CA125 among five other biomarkers, require further validation [7]. Although these tests demonstrate that a combination of multiple markers can generate synergistic advantages over a single marker in a clinical setting, they are primarily based on upregulation of CA125, which does not always occur. Also, these tests are mostly used for further evaluation of women who have already been diagnosed with pelvic mass and are due for surgery, rather than for initial diagnosis. There are no current FDA-cleared biomarkers for OVC screening; markers are cleared only for the limited application of monitoring disease recurrence and therapeutic response.

Proteomics research over the last two decades has identified hundreds of potential biomarkers for OVC [8], and subsequently, appropriate validation methods have identified biomarkers with high sensitivity and specificity for early detection of the disease (Table 1). We have used a bioinformatics-guided approach to pinpoint a set of potential biomarkers for OVC. Subsequent screening and validation have distinguished three biomarkers with clinical relevance: human kallikrein 6, kallikrein 7, and PRSS8 [9–11]. Overexpression of these proteins is highly specific for OVC but not other cancer types; these proteins are significantly overexpressed in OVC cells, and are secreted into bodily fluids. We have recently published the potential of KLK6 and KLK7 as early detection biomarkers [12]. The current study provides data and analysis that define the potential use of PRSS8 as a biomarker for early detection of OVC.

**Table 1:** Biomarkers with high potential for early screening and diagnosis of OVC

Gene list									
ATP7B	**CA125**	CLEC3B	**KLK6**	TOP2A	ARID4B	CEA	ID2	IGFBP2	INHA
PDGFA	HE4	**DUSP1**	BSG	CLDN3	REEP5	MIF	IGF2BP1	LGALS3BP	CDC25C
BRCA2	CA72-4	**IL13RA2**	STAT3	CLDN4	CCT3	AFP	IQGAP1	MSLN	NME1
DNAJC15	BARD 1	PLK1	RAET1E	COPS5	CD47	PRL	RHOC	ST14	AKT2
KLK14	**BCL2**	VIL2	TITF1	CSF1	ETV4	MUC 1	RNASE2	AMH	ANGPT2
KLK9	IGFII	**APOD**	TFF1	EFNB1	MAGEA4	AMH	SYCP1	CDC25A	XIST
WFDC2	BAG1	CD247	SPINK1	KLK11	SCGB2A1	WT 1	TRIM25	CSF1R	KLK10
ERCC1	BAG3	CDC25B	**PRSS8**	KLK13	SIX5	OGP	**P11**	GADD45A	KLK15
KLK8	BAG 4	**DAB2**	CCNE1	MVP	ZNF217	**CDX2**	CYP2A	HLA-G	KLK5
RBL2	OPN	HMGA1	CEACAM6	PARP1	EYA2	SMRP	PTK2	JUP	**KLK7**
SKP2	Maspin	HOXB7	ETS1	**VEGFC**	ELF1	Bcl-xL	TACC3	MLANA	SOD2
**IGFBP5**	MSN	BCHE	EPHA2	ASNS	MUC5AC	TNFRSF1B			

Genes that were differentially regulated in OVC were identified by mining the Cancer Gene Index (CGI) by BioXM software. The search identified 117 differentially regulated genes. Of these, 13 genes were identified that exhibited major differential expression in 19 OVC cell lines vs. normal ovarian cell lines (*P* < 0.05 in all comparisons) (underlined and bold type)

Human prostasin, a trypsin-like proteinase (40 KDa), is a glycosyl-phosphatidyl-inositol (GPI)-anchored extracellular serine protease. It is encoded by PRSS8, which is located on chromosome 16p11.2. Prostasin is also known as Channel Activating Protease 1. It was first isolated from seminal fluid, and is normally produced by the prostate gland. It is expressed in epithelial cells and ducts of the prostate [13]. It is also present in low levels on the apical surface of epithelial tissues such as lung, kidney, liver, bronchi, colon, and salivary glands, indicating that it may have roles in multiple biological processes [11]. Prostasin is present in multiple tissues that absorb sodium [14]. It acts as a proteolytic activator of the epithelial sodium channel in vitro, and plays a major role in regulating sodium balance [15–17]. Aberrant expression of prostasin is associated with many cancer types such as urinary bladder, uterus, prostate and ovarian, as compared to its level in corresponding normal tissue [18–20]. However, activation of epithelial sodium channels by prostasin suppressed in vitro invasiveness of both prostate and breast tumor cells [13, 21, 22]. Loss of PRSS8 in bladder cancer is associated with epithelial to mesenchymal transition – a process during which epithelial cells are converted to migratory and invasive cells [23]. However, in OVC, it is difficult to deduce the role of PRSS8 based on its expression. The levels of PRSS8 in ovarian carcinoma cell lines are elevated, and the protein level is increased in the serum of late stage OVC patients. It was suggested that prostasin cleaves the extracellular domain of epidermal growth factor on epithelial cells; consequently, the receptor remains continuously phosphorylated and can potentially trigger metastasis [11].

Serum prostasin was measured by microarray technology in 64 OVC patients and in 137 normal individuals [24]. The serum prostasin mean level of detection was 13.7 μg/ml in OVC patients compared to 7.5 μg/ml in control subjects. Sensitivity and specificity of PRSS8 as a biomarker was calculated as high as 92 and 94 %, respectively. Moreover, post-operation levels of PRSS8 declined significantly in the majority of patients, indicating that PRSS8 may be potentially used not only as a diagnostic but also as a prognostic biomarker [24]. Similarly, levels of PRSS8 mRNA were evaluated in 12 OVC patients and normal prostate tissues by RT-PCR and immunostaining [11]. It was found that PRSS8 levels were 120 to 410–fold higher in OVC patients than normal controls [11]. PRSS8 levels in OVC cell lines were shown to be linked to regulation by zinc-finger protein 217 (ZNF217). This protein is commonly overexpressed during cancer progression, and promotes tumor cell survival. Silencing of the ZNF217 gene in the OVC cell line HO-8910 resulted in a nearly 8-fold down-regulation of 164 genes including PRSS8. WFDC2 (HE4), which is currently used as an early detection biomarker for OVC, was also found to be downregulated [25]. Results from these studies placed PRSS8 on the list of potential biomarkers for early detection of OVC [26].

Our study presents evidences to demonstrate that PRSS8 can be used as an early detection biomarker for OVC. CA125 is a common OVC biomarker used in the clinic; however, as discussed above, although it is widely expressed on tumor cells, CA125 demonstrates low sensitivity. However, in combination, CA125 and PRSS8 increased the sensitivity to 92 % and specificity to 94 % [24]; whereas, sensitivity of CA125 and PRSS8 alone was 64.9 and 51.4 %, respectively. In the same study, it was also shown that there is a low correlation between expression of CA125 and PRSS8, which is consistent with their function in different pathways; therefore, as biomarkers, they may be complementary. In our recent review, we indicated that CA125 and PRSS8 signal through multiple signaling pathways, including PI3K, AKT, ERK [27]. It will, therefore, be interesting to investigate the mechanism of the synergistic effect of PRSS8 on CA125 as an early detection biomarker. The results of our current study indicate that PRSS8 is absent in normal ovarian tissues at the gene and protein level, and is upregulated from very early stages of the disease. Thus, PRSS8 exhibits properties of a complementary biomarker to CA125 for early detection of OVC.

## Methods

### Ethics approval

The continuing review # CR00003202 for the ovarian study protocol # Pro00002901 was approved by the Institutional Review Board (IRB).

### Cell culture

A library of OVC cell lines and corresponding normal ovarian cells were obtained and cultured in specified conditioned media as described previously [12]. Briefly, OVC cells lines used were TOV112D, OV-90, CAOV3, SKOV3, PA-1, SW626, and ES-2 (ATCC, Manassas, VA); SKOV-1, IGROV-1, HEY, A2780, and 2008 (S. Howell, UCSD); UCI-101 and UCI-107 (P. Carpenter, UCI); DOV-13 (R. Bast, MD Anderson Cancer Center, TX); 2774 (J. Wolf, MD Anderson Cancer Center, TX); BG-1 (Dr. K. Korach, NIH, NC); normal ovarian epithelial cell lines FHIOSE118 (J. Cheng, Moffitt Cancer Center, FL) and IOSE523 (N. Auersperg, University of British Columbia, Canada).

### Immunoblot

Serum samples were depleted of abundant proteins by Affinity column ProteoPrep Blue Albumin and IgG Depletion Kit as described by the manufacturer (Sigma-Aldrich, St. Louis, MO). Protein concentrations were determined by Bradford Assay (Bio-Rad Laboratory, Hercules, CA), and 20 μg of total protein was resolved by 12.5 % SDS-PAGE for immunoblot analysis. Sera from OVC patients were purchased from Proteogenex (Culver City, CA) and Bioserve (Beltsville, MD). Nine normal sera were pooled to represent normal donors. A custom-made PRSS8 antibody (Precision Antibodies, MD), HRP-conjugated secondary antibodies (Jackson ImmunoResearch Laboratories, West Grove, PA) and SuperSignal West Dura (Thermo Fisher Scientific, Rockford, IL) were used for visualization of protein bands.

### Immunohistochemistry and In situ hybridization

Whole mount tissues and tissue arrays containing different stages and subtypes of OVC were purchased from US Biomax (Rockville, MD) and Proteogenex. Tissue sections were de-paraffinized using Histochoice clearing agent (Amresco, Solon, OH) for 5 min followed by hydration steps with 100, 90, 70, and 50 % ethanol for 5 min each. After equilibrating with PBS for 5 min, the tissues were incubated with high pH solution (Amresco) at 95 °C for 20 min to retrieve antigens. The sections were cooled and washed with PBS for 5 min, and endogenous peroxidases were blocked by incubating in 3 % H_2_O_2_ for 15 min. The sections were marked with a hydrophobic PAP pen (Vector Labs, Burlingame, CA), blocked for 30 min at 37 °C in 5 % BSA in PBS/0.1 % Tween-20, and then incubated with the primary antibody (SC-136272, Santa Cruz Biotechnology) overnight at 4 °C. The sections were washed thrice in PBS/0.1 % Tween-20 for 5 min each. The tissues were incubated with secondary antibody (715–506-151 Jackson Immuno Research Laboratories) for 30 min at RT and washed as above. A DAB kit (Vector Labs) was used to visualize the antigen. Color development was interrupted by washing with distilled water for 5 min. Hematoxylin (Amresco) was used as the counterstain. Sections were dehydrated in ethanol solutions in the sequence of 50, 70, 90, 100 for 5 min each and 5 min in Histochoice clearing agent. After mounting the tissues (Permount, Vector Labs), the stained tissues were photographed using an Axio Imager Microscope (Carl Zeiss, Thornwood, NY).

In situ hybridization was performed as we previously described [12]. The probe sequence for PRSS8 was; 5’- DIGN-GCAGTAAAACTCCTGACTCTCA.

### qRT-PCR

Total RNA was extracted from cells using Trizol (Invitrogen, Carlsbad, CA), and cDNA was generated with the SuperScript III RTS First-Strand cDNA Synthesis Kit (Invitrogen). All primers were custom synthesized to be used with an ABI7900 RT-PCR instrument (Applied Biosystems, Foster City, CA) as recommended by the manufacturer. Primers were further validated using end-point PCR of cDNA generated from the normal ovarian cell lines; all primers produced a single band with the expected size as visualized on an agarose gel. The primers were typically 20-mers having a T_m_ of 58 °C. For qPCR, 43 ng cDNA, 10 pmole primers, and SYBR Green PCR Master Mix (Applied Biosystems) were combined in a 20 μl reaction volume. All qPCR were performed in MicroAmp Fast Optical 96-Well Reaction Plates with Barcode (Applied Biosystems) in the standard mode (1^st^ denaturation at 95 °C for 10 min followed by 40 cycles of 95 °C for 15 s and 60 °C for 1 min). The qPCR data were normalized with GAPDH, and further analyzed using software provided with the ABI7900. TissueScan Cancer Survey Panels (OriGene, Rockville, MD) were used for screening 22 different human cancer types (over 380 biospecimens), and Ovarian Cancer Panels I-IV (OriGene) were used for determining the expression levels of genes at various stages, grades, and subtypes of OVC (over 190 biospecimens). TissueScan Cancer Survey Panels were purchased in a 96-well format with lyophilized cDNA from various patients with different cancer types. Each well of the plate contained 2–3 ng of cDNA, and the plate was divided to scan two genes. The reaction mix was transferred to a ‘Fast Plate’, compatible with the ABI 7900 HT RT-PCR instrument. After dividing each plate into two ‘Fast-Plates’, each reaction consisted of approximately 1–1.5 ng of cDNA. The conditions used were: 1^st^ denaturation at 95 °C for 10 min followed by 40 cycles of 95 °C for 15 s and 60 °C for 1 min. The data from TissueScan panels were normalized with beta-Actin. All cancer tissues used in these panels contained an average of 75 % cancer cells and 25 % surrounding stromal components.

## Results

### Identification of PRSS8 as a potential biomarker for OVC

**Fig. 1:**
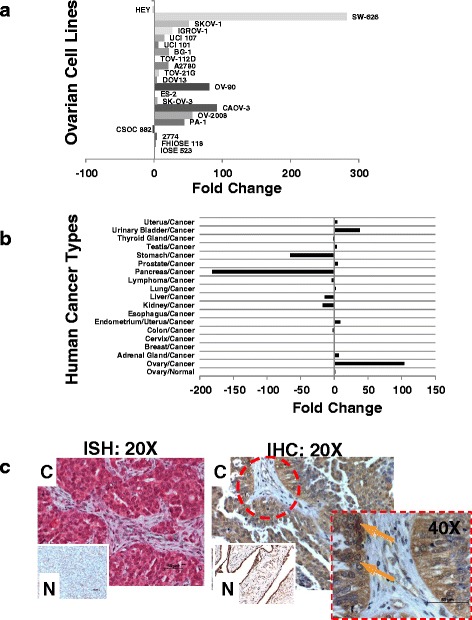
Expression of PRSS8 is specific to OVC cells. Expression of PRSS8 in normal and OVC cell lines (**a**), in tissue of normal ovary, OVC, and other types of cancer (**b**), and as detected in ISH (C – *left*) and IHC (C – *right*) in normal (N – in bottom left corner of ISH and IHC) and OVC tissues (magnification 20X and 40X). **a** Gene expression of PRSS8 in OVC cell lines was analyzed by qRT-PCR and normalized. Fold change represents the level of gene expression in OVC cell lines normalized against a normal ovarian cell line IOSE523. **b** Fold change represents the level of gene expression in cancer tissue samples normalized against the corresponding normal tissue; here, *p* < 0.001, PRSS8 expression in OVC vs. other cancer types; one-way ANOVA (SigmaStat). **c** (ISH) *left*, mRNA of tissues of ovarian tumors in normal individuals and OVC patients were hybridized In situ (20X magnification). (IHC) *right*, Detection of prostasin levels by immunohistochemistry. Nuclear stain is indicated with solid arrows

Multiple “-omics” studies in the past two decades have produced more than 5000 publications related to biomarkers for OVC [28]. To identify biomarkers with the greatest potential for utility in population screening and early detection, the BioXM bioinformatics platform was used to search the Cancer Gene Index database of the National Cancer Institute (USA) with query strings including “ovarian, cancer, biomarker, upregulation, downregulation, differential expression, and overexpression.” The output data set (Table 1) contained 117 genes representing multiple signaling pathways including pathways related to apoptosis, proliferation, and angiogenesis. For the screening process, a library of 19 OVC cell lines analyzed (Table 2); eventually, 13 genes that were differentially regulated in more than 70 % of the cell lines were selected. Among these genes, BCL2, CDX2, KLK7, KLK6, P11, PRSS8, and CA125 were upregulated, and IGFBP5, DUSP1, DAB2, VEGFC, IL13RA2, and APOD were downregulated. A real-time qPCR analysis showed that PRSS8 was upregulated in the majority of OVC cell lines compared with two normal ovarian epithelial cell lines (FHIOSE118 and IOSE523, Fig. 1a). Using a similar approach, we recently reported that KLK6 and KLK7 are potential early detection biomarkers for OVC [12].

**Table 2:** Ovarian cell lines used in biomarker screening

Cell line	Source	Tumor histology	Grade	Stage	Derived from	Oncogene
OV-2008	Dr.Stephen B.Howell, UCSD	Endometrioid carcinoma	-	-	Tumor	N/A
2774	Dr.Judith Wolf, MD Anderson Cancer Center, Texas	Endometrioid carcinoma	2		Ascitic fluid	N/A
A2780	Dr.Stephen B. Howell, UCSD	Ovarian carcinoma	-	-	Tumor	N/A
BG-1	Dr.Korach, NIH	Adenocarcinoma	-	II	Tumor	N/A
CAOV-3	ATCC	Adenocarcinoma	-	-	Tumor	FAM123B, STK11, TP53
CSOC 882	Dr. Karlan, UCLA	Adenocarcinoma	3	IC	Tumor	EGFR, HER2,
DOV13	Dr.Robert Bast JR., MD Anderson Cancer Center	Adenocarcinoma	-	-	Ascites	N/A
ES-2	ATCC	Clear cell carcinoma	3	-	Tumor	P glycoprotein
HEY	Dr.Stephen B.Howell, UCSD	Adenocarcinoma	-	-	Xenografted ovarian tumor	KRAS + BRAF
IGROV-1	Dr.Stephen B.Howell, UCSD	Adenocarcinoma	2	III	Tumor	N/A
OV-90	ATCC	Malignant papillary serous adenocarcinoma	3	IIIC	Ascites	her2/neu +, p53
PA-1	ATCC	Teratocarcinoma	-	-	Ascites	N-ras + (activated)
SKOV-1	Dr.Stephen B.Howell, UCSD	Clear cell carcinoma	-	-	Ovary	N/A
SK-OV-3	ATCC	Adenocarcinoma	-	-	Ascites	MLH1, CDKN2A, TP53,PIK3CA
SW-626	ATCC	Adenocarcinoma	-	-	Ovary	N/A
TOV-112D	ATCC	Malignant adenocarcinoma	3	IIIC	Tumor	her2/neu +, p53
TOV-21G	ATCC	Malignant adenocarcinoma	3	III	Tumor	p53+ (WT)
UCI 101	Dr.Philip Carpenter, UCI	Papillary Seous Adenocarcinoma	-	III	Ascites and Tumor	p-glycoprotein, EGFR
UCI 107	Dr.Philip Carpenter, UCI	Papillary Adenocarcinoma	-	III	Tumor	N/A
FHIOSE 118	Dr.Cheng, Moffit Cancer Center,	Immortal normal ovarian surface epithelium	-	-	-	N/A
IOSE 523	Dr.Nelly Auersperg, University of British Columbia, Canada	Normal ovarian epithelium	-	-	-	N/A

Nineteen cell lines that were generated from OVC patients and two cell lines that were generated from normal ovaries were used in this study. IOSE523 is a “primary-like” normal ovarian cell line. IOSE523 cells begin to senesce after 20 passages while FIOSE118 cells are immortal

### PRSS8 overexpression is substantially specific to OVC

PRSS8 expression was analyzed in cancer tissue and corresponding normal samples from 18 major human tumor types from 390 individuals (TissueScan from Origene, data not shown). The PRSS8 gene was overexpressed in the majority of cancer types tested. In OVC, PRSS8 showed a 106-fold overexpression compared to expression in epithelial cells of normal ovaries (Fig. 1b). The PRSS8 gene was overexpressed 47-fold in urinary bladder cancer, but was downregulated in pancreatic cancer (180-fold) and in stomach cancer (75-fold) (Fig. 1b). In an analysis of nearly 500 ovarian tumors and normal ovarian tissues by In situ hybridization and Immunohistochemical staining in Tissue Array format, we found that the PRSS8 gene and PRSS8 protein (prostasin) were both significantly upregulated in the majority of ovarian tumors (Fig. 1c). PRSS8 transcripts were expressed at a low basal level in normal ovaries, but expression increased significantly in the epithelial compartment of OVC tumors (Fig. 1c, ISH). In all tumor subtypes examined, the neighboring stromal compartment exhibited low basal level staining, suggesting predominant expression of PRSS8 in tumor epithelium. Similarly, the immunohistochemical staining of prostasin was identical to the In situ staining pattern (Fig. 1c, IHC), and positive staining was observed in cytoplasm and nucleus of tumor epithelium. The neighboring stroma adjacent to tumor epithelia were minimally stained in all OVC tissues tested (Fig. 1c).

### The PRSS8 gene is overexpressed in early stages and grades of OVC

**Fig. 2:**
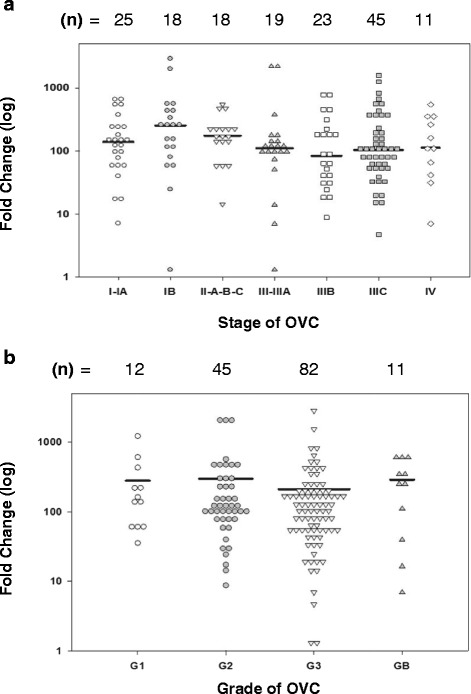
PRSS8 expression is upregulated throughout all stages of OVC. **a** PRSS8 gene expression levels were measured in tumor tissues of OVC patients at different stages of the disease, and were plotted as individual fold increases. **b** Average levels of PRSS8 in tumor tissues of OVC patients presented as a function of disease grade

PRSS8 gene and prostasin expression were analyzed in biospecimens derived from all stages and grades of OVC malignancy. OVC staging involves a determination of metastatic potential by examining cells and tissues collected from distal sites. Stage I represents OVC tumor that is confined to one or both ovaries. At Stage II, OVC cells are already metastatic, and localize to other sites in the pelvic area, including uterus and fallopian tubes; this early metastatic characteristic makes OVC the deadliest gynecological cancer. When OVC reaches Stage III, tumors spread to abdominal organs and lymphatic compartments. In Stage IV, metastasis spreads to distal organs (lung, liver, brain). In our experiments, each stage was divided into A, B, and C categories. OVC biospecimens were grouped into seven categories: Stage I-IA (*n* = 25), IB-IC (*n* = 18), IIA-B-C (*n* = 18), III-IIIA (*n* = 19), IIIB (*n* = 23), IIIC (*n* = 45) and IV (*n* = 11) (Fig. 2a). We found that expression of the PRSS8 gene was upregulated in I-IA, I-IB, and II-A-B-C stages. The mean level of overexpression (>100 fold) was maintained throughout all OVC stages (Fig. 2a). Similarly, when we examined different grades of OVC, the mean level of PRSS8 overexpression was nearly 300-fold, regardless of grade (Fig. 2b). Due to differences in the number of patients tested in each group, un-paired t-tests were performed; there were no significant differences in PRSS8 expression among the groups of patients either at different stages (*P* > 0.05) or in different grades (*P* > 0.05). The data from both early stages (I/II) and grades (low) suggest that PRSS8 might provide an OVC early detection biomarker. Significant statistical differences were not found in the mean values of PRSS8 expression across all OVC stages and grades, suggesting that PRSS8 would not serve to differentiate OVC stages and grades (Fig. 2).

### Overexpression of PRSS8 in OVC is subtype-dependent

**Fig. 3:**
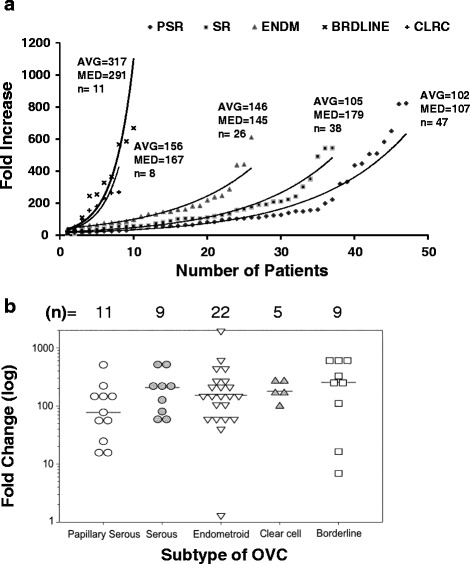
PRSS8 expression is elevated in different types of OVC. **a** Levels of PRSS8 were measured in all stage groups of OVC patients and are presented as fold increase over expression in normal individuals. **b** Expression of PRSS8 in early stage patients (stages I and II) in groups representing 5 different OVC subtypes. Results are presented as fold change compared to expression in normal individuals. OVC subtypes are as follows: papillary serous (PSR), serous (SR), endometriod (ENDM), borderline (BRDLINE), clear cell (CLRC)

Ovarian carcinoma subtypes exhibit differences in tumor initiation and progression. Survival analyses on a large cohort of OVC showed that only 1 of 3 potential OVC prognostic biomarkers was meaningful with respect to specific OVC subtypes [29]. To determine whether the signature of PRSS8 expression differs among OVC subtypes, five major OVC subtype tumor samples were analyzed. We found significant differential expression of PRSS8 among OVC subtypes (Fig. 3a). The borderline subtype exhibited the greatest upregulation (291-fold over normal control); this was followed by serous (179-fold), clear cell (167-fold), endometrial (145-fold), and papillary serous (107-fold). Because of the low numbers of samples for each of the subtypes, it was not possible to perform rigorous statistical analysis; however, the median trend lines indicated that the variation in PRSS8 expression among subtypes was likely cell type-dependent (Fig. 3a). When early stage OVC tumors (stage I and II) were selectively analyzed, all subtypes exhibited a similar level of PRSS8 upregulation (approximately 100-fold; *p* > 0.05), suggesting that variation in expression levels among subtypes was largely contributed by stage III and IV tumors (Fig. 3b).

### Prostasin expression is absent in normal ovary but frequently abundant in benign and OVC tumors

**Fig. 4:**
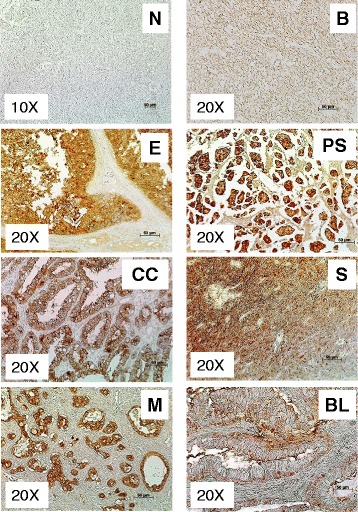
Localization of prostasin in OVC, benign, and normal ovarian tissues. Tissue arrays of OVC, benign, and normal cases were stained for prostasin by immunohistochemistry (magnification of 10-20X). Tissue arrays were generated from normal ovary (N), benign ovary tissue (B), endometriod adenocarcinoma (E), papillary serous adenocarcinoma (PS), clear cell carcinoma (CC), serous adenocarcinoma (S), mucinous adenocarcinoma (M), and borderline carcinoma (BL)

**Fig. 5:**
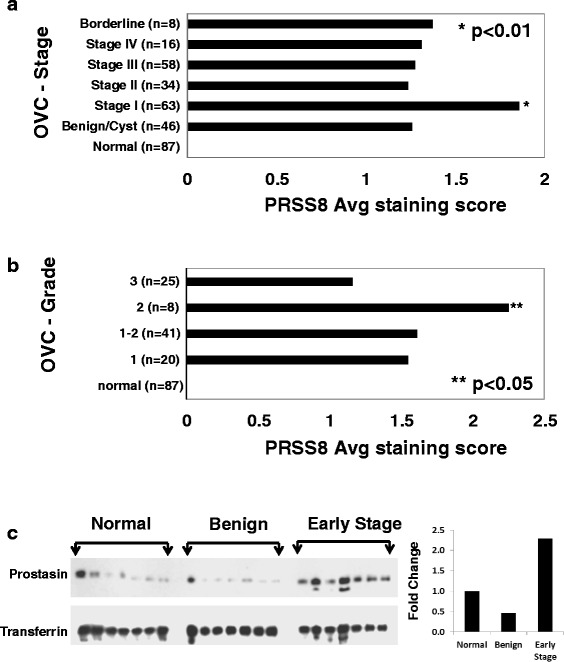
PRSS8 is upregulated in tissues of OVC patients compared to benign and normal tissues. Bar plots of PRSS8 immunostaining score by OVC stage (**a**) and OVC grade (**b**); *n* = number of stained arrays in each group. Immunostaining of all tissue arrays used in this study was score (range: 0–3) according to levels of staining, where score of (0) means negative staining, (1)-weak positive staining, (2)-positive staining, (3)-strong positive staining. Serum samples from OVC (early-stage), benign, and normal subjects (7 in each group) were subjected to western blot for the appearance of PRSS8 and densitometry values for each group were plotted (**c**). Primary PRSS8 antibody used in this study is a custom-made antibody (see details in Additional file 1)

We determined correlations between PRSS8 overexpression and protein expression in early stage OVC tissues by immunohistochemical analysis. Staining intensity on 312 tissues representing normal ovary, benign mass, and OVC tissues in tissue-array format were qualitatively scored based on pathological guidelines (Fig. 4). Prostasin staining was negative on normal ovary tissue sections (Fig. 4N) and was similar to the negative control (Additional file 1). However, prostasin was abundantly present in early stage OVC subtypes (Fig. 4B, E, PS, CC, S, M, BL); staining was similarly positive in all late stage OVC (data not shown, see scores in Fig. 5). For benign ovarian tissues (theca cell tumors and simple cysts; *n* = 46), the staining intensity was similar to OVC tumors (data not shown). In all benign and OVC cases tested, prostasin was abundantly present in serous, papillary serous, mucinous, endometriod, clear cell, borderline, and transitional OVC tumors (Fig. 4). When non-OVC tissues such as omentum and mixed mullerian tumor mass were examined, minimal or no staining was observed (data not shown), suggesting that prostasin expression may be specific to the epithelial origin of ovarian tissues. For qualitative analyses, groups of stained normal ovary, benign, and OVC subtype tissues were visually scored for intensity (from 0, no staining, to 3, intense staining) on five random fields under a bright field microscope. The average scores indicated that prostasin was abundantly present in benign and early stages (*p* <0.01) and low grades (*P* < 0.05) of epithelial OVC compared to normal ovary tissues (Fig. 5a and b). The expression of prostasin was consistent with the PRSS8 gene overexpression data, suggesting that expression of either PRSS8 (at the transcriptional level) or prostasin (at the histologic level) are robust biomarkers suitable for early detection of OVC.

**Table 3:** Immunohistochemical analysis as a function of age of subject

		Normal (young adults)		Normal (>30 year old)		Benign		OVC	
		(−)	(+)	(−)	(+)	(−)	(+)	(−)	(+)
Age	AVG	19.9	0	50.7	51.9	56.2	52.2	53.7	51.6
	MAX	21	0	70	70	80	77	78	75
	MIN	18	0	34	32	16	22	34	19
	n	11	0	18	12	24	15	20	29
	STD	1.2	0	11	10.6	17.2	14.8	13.3	12.7
						
	t			>0.05 (t = 0.38)		>0.05 (t = 0.5)		>0.05 (t = 0.29)	

Tissue samples from healthy subjects, patients with benign lesions, and OVC patients were tested by immunohistochemistry for the appearance of PRSS8. Correlation analysis between PRSS8 and age of subject was performed

### Prostasin level is elevated in serum of early-stage of OVC

It is preferable to screen patients for biomarkers found in serum as blood collection is minimally invasive and is routinely performed. To determine whether prostasin was secreted into the circulation and whether it could be detected in early phase (I/II) OVC, we performed immunoblot analysis on serum samples from benign OVC, OVC-I/II, and normal donors (Fig. 5c). Abundant protein-depleted sera (see Methods) were analyzed by in-house anti-prostasin antibody (see Additional file 1) made against a prostasin-specific N-terminal peptide. This antiserum was highly specific and was effective at 10 pg/ml for immunoblotting prostasin. We found that the mean prostasin level was more than two fold higher in serum samples from early stage OVC patients than from benign or normal controls (Fig. 5c).

## Discussion

Ovarian cancer causes the death of over 125,000 women worldwide each year, which is more than all other gynecologic cancers combined. Women visiting the clinic with apparent symptoms are usually categorized with late stage (III-IV) OVC. Less than 20 % of all reported OVC cases are diagnosed in early stages, primarily because of the complexity of the disease and lack of specific biomarkers. In this report, we show that PRSS8 is a potential biomarker that is up-regulated in OVC at all stages, grades, and major subtypes.

More than a hundred potential biomarkers for OVC have been identified via multiple “-omics” methods (Table 1). In our work, to simplify screening without using precious biomaterials from OVC patients, a library of 21 ovarian cell lines (Table 2) was used in this initial phase to screen candidate biomarkers. PRSS8 was identified based on its robust and consistent overexpression in the majority of those OVC cell lines (Fig. 1). This robust overexpression signature was further validated in OVC patient samples, where we found differential expression of more than 100 fold compared with normal epithelial ovary tissues (Fig. 1a). PRSS8 was also significantly upregulated in urinary bladder cancer but downregulated in pancreatic and stomach cancer, suggesting that the expression of PRSS8 in tumors may be related to the specific cell or tissue type of tumor origin. The overexpression of PRSS8 and the abundance of prostasin in OVC tissues at early stages and low grades showed that both are excellent candidates as early detection biomarkers. We have previously demonstrated that KLK6 and KLK7 can serve as ovarian cancer-specific biomarkers. These also exhibited selective upregulation in OVC (12). It is likely that a combination of PRSS8, KLK6, and KLK7 can provide additional specificity and sensitivity for early detection of OVC.

The absence of PRSS8 and prostasin in normal epithelium and stroma indicates that gene and protein expression are tightly regulated in non-cancerous tissues. The significant overexpression profile at the onset of OVC and maintenance of this signature throughout OVC progression suggest that prostasin function may be required for maintaining the OVC phenotype (Fig. 2). We did not find significant differences between different stages and grades of OVC in the samples tested, indicating that PRSS8 and prostasin can be used as screening biomarkers for every stage and grade, including late stages and high grades, of most OVC subtypes. PRSS8 overexpression in borderline OVC may indicate that PRSS8 can also be used to detect low-incident OVC subtypes (<15 % in US). Median levels of PRSS8 gene expression were highest in borderline and clear cell OVC, followed by serous, papillary serous, and endometriod subtypes, indicating that PRSS8 expression is cell type-dependent within OVC subtypes (Fig. 3). We observed a robust overexpression profile of the PRSS8 gene in all OVC subtypes. The median overexpression was more than 100 fold suggesting that PRSS8 is an excellent candidate for early detection of OVC. The PRSS8 overexpression profile was largely maintained and translated into high protein expression in all stages and grades of OVC (Fig. 4), indicating that PRSS8 and prostasin can be used in OVC biopsy and small size samples. Using the combination of PRSS8/prostasin, KLK6, and KLK7 may provide a valuable diagnostic tool applicable for use on small OVC tissue samples available from clinical procedures. An additional analysis was that age does not influence upregulation of PRSS8 across the normal, benign and OVC tissue samples (Table 3). This further contributes to the overall strength of PRSS8 as a universal biomarker for early detection of OVC.

The immunohistochemical analysis in this report indicates that prostasin is downregulated not only in normal ovaries but also in several types of cancerous tissues that are in close proximity to the ovaries, such as the omentum and the uterus. In tissues tested, prostasin detected in normal and benign tissues was located in the membrane, but in OVC tissues prostasin was localized in the cytoplasm and nucleus, suggesting that cellular translocation of prostasin may be involved in OVC progression. In these test settings, the majority of benign tissues analyzed were of theca cell tumors (data not shown). Theca cells are endocrine cells that play an important role in fertility by producing androgen substrates that are key to estrogen biosynthesis [30]. Endocrine infertility is commonly caused by excessive proliferation of theca cells and ovarian hyper-androgenism, indicating that PRSS8 levels may be affected by hormonal changes and balance. In a genome-wide study aimed at identifying estrogen response elements (ERE), it was shown that these elements are also found in the coding sequence of PRSS8; the presence of a high-affinity binding site for estrogen suggests that estrogen may control PRSS8 expression [31], thus, elevating the level of prostasin in tissues. In a recent study, a regulatory network analysis of the estrogen receptor in a model of renal cell carcinoma indicated that estrogen may be involved in regulation of oncogenes and tumor suppressor genes, including PRSS8 [32].

Although the cohort was small, our analysis of serum indicated that prostasin was largely absent in normal donor sera and benign OVC serum samples but was frequently abundant in OVC serum samples. In our analysis, samples that showed positive results were derived from thecoma patients, and the role of prostasin in benign ovarian samples is not well determined. PRSS8 was previously suggested as a potential biomarker for OVC at benign stages [33], and our study further validated these findings. We would also emphasize that early detection methods for screening the general population should be non-invasive or minimally invasive method because the population without clinical symptoms most likely will not participate in any invasive clinical procedures. Blood tests are ideal for screening of asymptomatic patients during routine clinic visits. prostasin is a known secreted protein, and is detected in multiple human biological fluids, including peripheral blood, and thus, would be an excellent candidate as a serum biomarker for early stage OVC.

Earlier studies demonstrated upregulation of PRSS8 in early stages of OVC [9, 21], but some reports showed conflicting data [34, 35]. The abundance of prostasin in serum of OVC patients showed significant potential to be used as OVC biomarker but required strict maintenance of standardized conditions for accurate analyses [33, 36]. Prostasin level does not change in urine before and after menopause; oral contraceptives or estrogen and progesterone therapy tend to increase PRSS8 levels albeit not significantly [37]. However, an inaccurate diagnosis may be made in circumstances where abnormal hormone levels occur but are associated with stress. In a study where a large cohort (*n* = 500) of OVC samples was assessed [33], the need for standardized conditions was emphasized. In that study, prostasin was one of nine selected OVC serum biomarkers, and presented the highest discriminatory value (*P* < 0.001) compared to benign cases. Similarly, our normal donor controls were procured during typical routine ‘clinical visits’. To further increase the sensitivity and specificity of prostasin detection, we generated a custom-made anti-prostasin antibody against prostasin (Additional file 1). The antiserum included a high titer antibody with high specificity to prostasin in serum samples derived from normal donors, benign, and OVC serum samples. Elevated prostasin levels in early stage OVC serum samples indicated that the prostasin secretion pathway was active, and that significant overexpression of the PRSS8 gene was translated to elevated prostasin levels in circulation. Thus, the abundance of prostasin correlates with overexpression of PRSS8 in early stage OVC. This correlation may be useful for initial population screening by prostasin, and for further clinical evaluation by PRSS8/prostasin analyses of ovarian biopsies.

Cancer Antigen 125 (CA125) is widely used in the clinic as a serum biomarker for OVC because it is elevated significantly in late stages. However, CA125 lacks specificity and sensitivity for early stage OVC as it detects less than 23 % of cases in stage I, while detecting greater than 80 % in late-stage OVC [38, 39]. CA125 is also frequently upregulated in benign conditions (e.g., endometriosis, fibroids, etc.) and during ovulation; thus, CA125 lacks accurate diagnostic value for early stage disease in pre-menopausal women. Human Epididymis Protein 4 (HE4) has better sensitivity and specificity than CA125 for early detection of OVC [40]. A combination of HE4, CA125, carcinoembryonic antigen (CEA), and vascular cell adhesion molecule (VCAM)-1 in an assay panel has been tested for detecting early stage OVC versus benign tumors, and achieved 86 % sensitivity [41]. OVA1 and other OVC biomarker tests represent an effort to increase statistical power of early detection of OVC. In a recent study, PRSS8 showed significant synergy for increasing sensitivity and specificity when it was combined with OVA1 and tested against tissue samples from all stages of OVC [33]. That study tested over 200 biomarkers (including CA125 and HE4) but none were sufficiently informative to be sole biomarkers for the broad applications and subtypes of OVC presented. Similarly, PRSS8 did not discriminate among OVC subtypes in our study, but the expression levels were significantly lower in clear cell and mucinous subtypes. In general, PRSS8 presented a poor correlation with CA125 and a moderate correlation with HE4 (*p* = 0.463), further supporting the idea that HE4 is a better early detection OVC biomarker than CA125. Both panels of the 5 (OVA1) and the 9 (including PRSS8) biomarkers performed better (as measured by AUC values) for post-menopausal women compared to pre-menopausal women [33].

A recent trend in early detection of OVC cancer is to measure longitudinal individual changes in levels of potential biomarkers [42]. For example, the United Kingdom collaborative trial of OVC screening (UKCTOCS) followed more than 200,000 women, 50-years old and older, and compared the impact of screening by detecting CA125 levels vs. ultrasound, and then correlated the findings with OVC disease outcomes. The data were encouraging, but the conclusion was that additional biomarkers should be added to CA125-based screening to achieve a better clinical outcome. Prostasin can potentially be used for population screening by serum testing. Prostasin-positive patients could be guided to the clinic for further evaluation, where PRSS8 gene levels and prostasin protein levels could be measured in ovarian biopsies for diagnosis. Based on our current data on PRSS8/prostasin and on our previous report on KLK6 and KLK7 [12] we believe that these members of the kallikrein family can potentially be combined with PRSS8/prostasin for early detection and screening for OVC. Future studies should include a large cohort of OVC tissues and serum samples to further validate the use of PRSS8 and prostasin, especially with KLK6, KLK7, HE4, OVA1, and CA125.

## Conclusions

The abundant amounts of secreted prostasin found in sera of early stage OVC can potentially be used as a minimally invasive screening biomarker for early stage OVC. Overexpression of PRSS8 mRNA and high levels of prostasin in multiple subtypes of early stage ovarian tumors may provide clinical biomarkers for early detection of OVC, which can potentially be used with CA125 and HE4.

## Additional file

Additional file 1: Figure S1. Characterization of custom-made PRSS8 primary antibody. Optimization of PRSS8 concentration by immunoblot (A). OD reading as a function of PRSS8 concentration as measured by Elisa (B). 1^st^ bleed and 2^nd^ bleed were normally taken on days 17 and 31, respectively, after rabbit immunization. Upper panel. Results from antibodies raised against N-terminus. Lower panel. Results from antibodies raised against C-terminus. **Figure S2.** Prostasin immunostaining in normal and OVC tissues. Insets: no primary antibody control. (PDF 573 kb)
