# Supplementary figures and images for: The serine protease prostasin (PRSS8) is a potential biomarker for early detection of ovarian cancer
Source: J Ovarian Res. 2016 Mar 31;9:20. doi: 10.1186/s13048-016-0228-9 (PMC4815131; doi:10.1186/s13048-016-0228-9)

Figure S1

A.

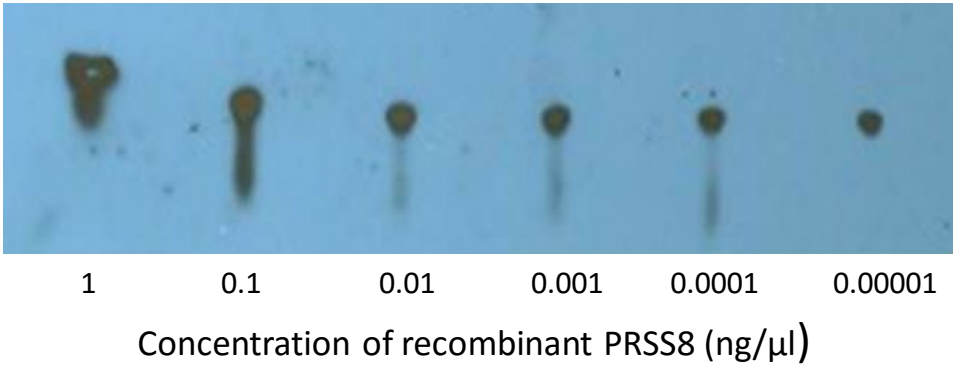

B.

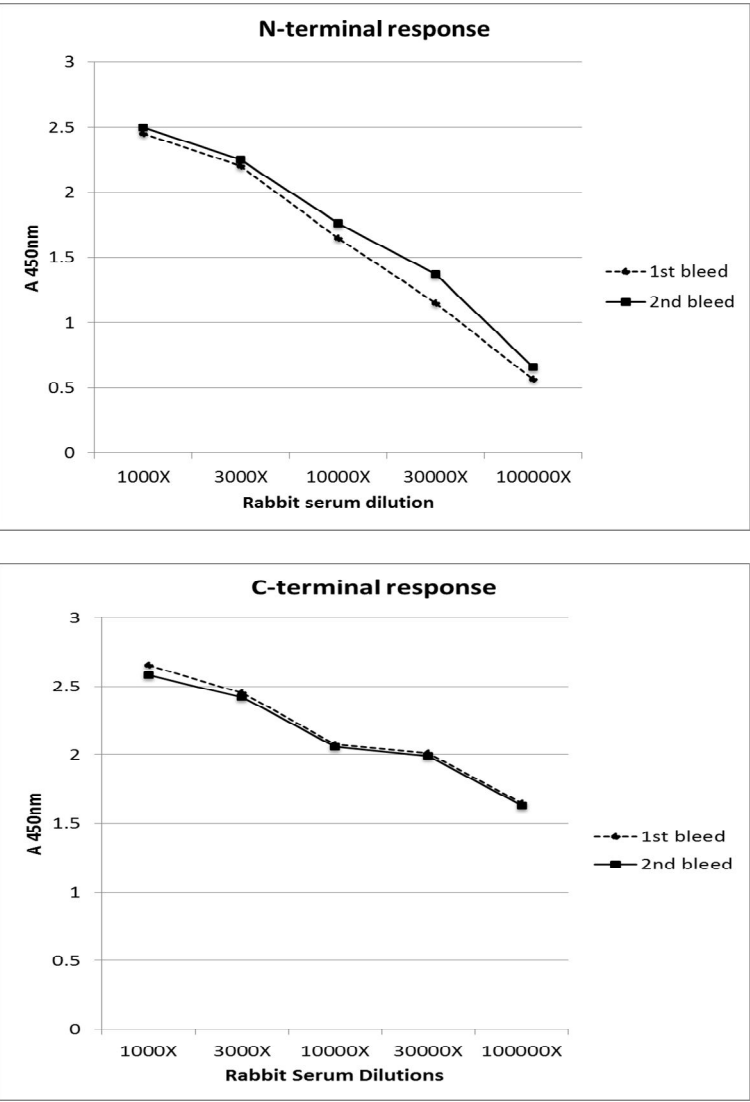

Figure S2

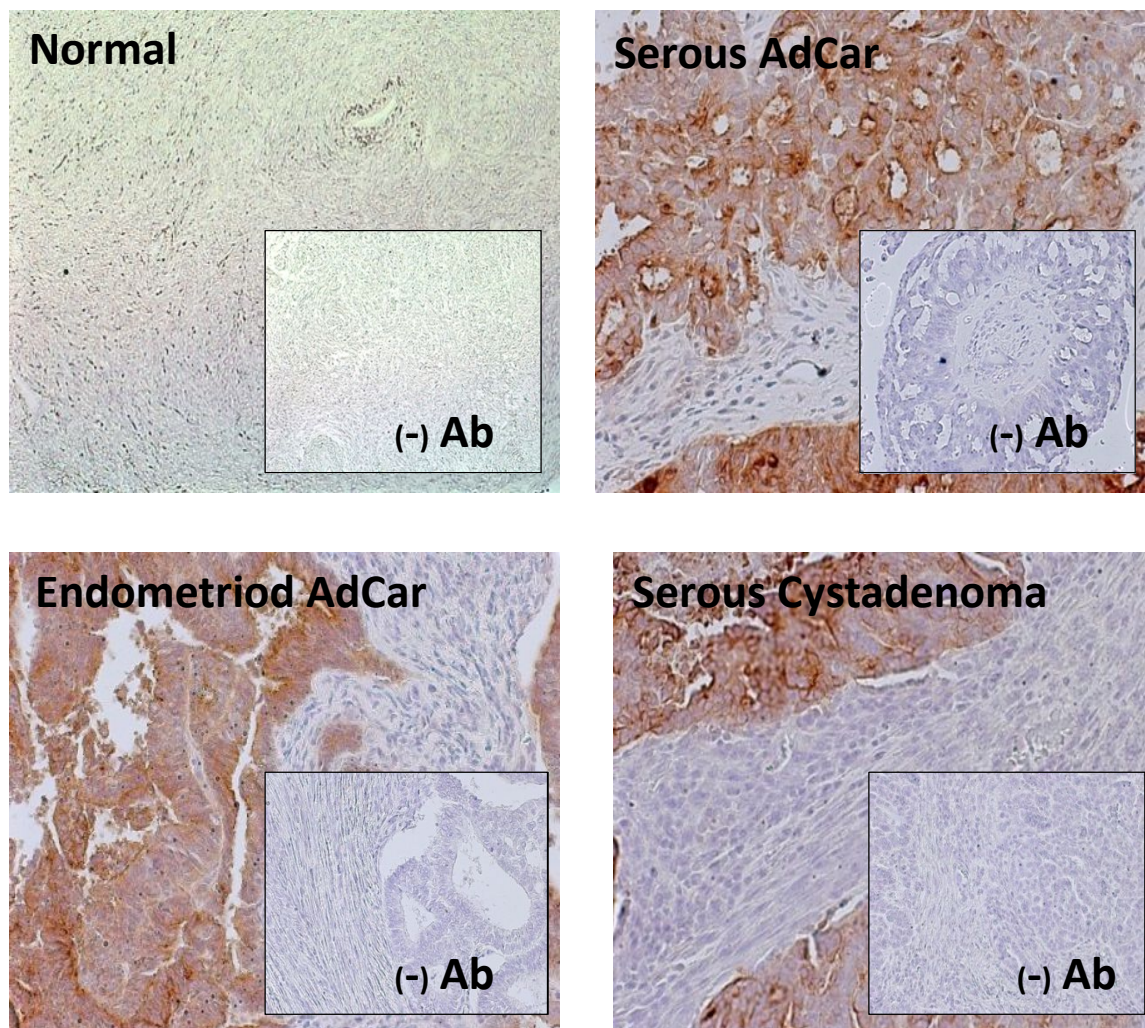

Supplement: Additional file 1: Figure S1. — Characterization of custom-made PRSS8 primary antibody. Optimization of PRSS8 concentration by immunoblot (A). OD reading as a function of PRSS8 concentration as measured by Elisa (B). 1st bleed and 2nd bleed were normally taken on days 17 and 31, respectively, after rabbit immunization. Upper panel. Results from antibodies raised against N-terminus. Lower panel. Results from antibodies raised against C-terminus. Figure S2. Prostasin immunostaining in normal and OVC tissues. Insets: no primary antibody control. (PDF 573 kb) [file 13048_2016_228_MOESM1_ESM.pdf]
